# Mechanisms of extracellular vesicle-mediated immune evasion in melanoma

**DOI:** 10.3389/fimmu.2022.1002551

**Published:** 2022-08-23

**Authors:** Lothar C. Dieterich

**Affiliations:** Institute of Pharmaceutical Sciences, Swiss Federal Institute of Technology (ETH) Zurich, Zurich, Switzerland

**Keywords:** exosome, immune checkpoint, immunotherapy, melanoma, lymph node, metastasis, tumor immunity, extracellular vesicle (EV)

## Abstract

Melanoma-derived extracellular vesicles (EVs) have been found to promote tumor growth and progression, and to predict patient responsiveness to immunotherapy. Consequently, EVs have been implicated in tumor immune evasion, and multiple studies reported immune-regulatory activities of melanoma EVs *in vitro* and *in vivo*. This review highlights mechanistic insights in EV-mediated regulation of various immune cell types, including effects on inflammatory, apoptotic, stress-sensing and immune checkpoint pathways as well as antigen-dependent responses. Additionally, current challenges in the field are discussed that need to be overcome to determine the clinical relevance of these various mechanisms and to develop corresponding therapeutic approaches to promote tumor immunity and immunotherapy responsiveness in melanoma patients in the future.

## Introduction

Extracellular vesicles (EVs) are membrane-encapsulated, subcellular particles that are released by virtually every cell type and have been ascribed various biologic functions, ranging from waste disposal to molecular cell-to-cell communication (1). Traditionally, EVs have been classified based on the membrane of origin and/or the mode of EV generation, including exosomes derived from endosomal membranes, microvesicles derived from the plasma membrane, and apoptotic bodies (2). However, due to difficulties to specifically isolate and distinguish between these EV subsets, a classification based on measurable, physical parameters (e.g. size, density) has been proposed (3).

**Figure 1:**
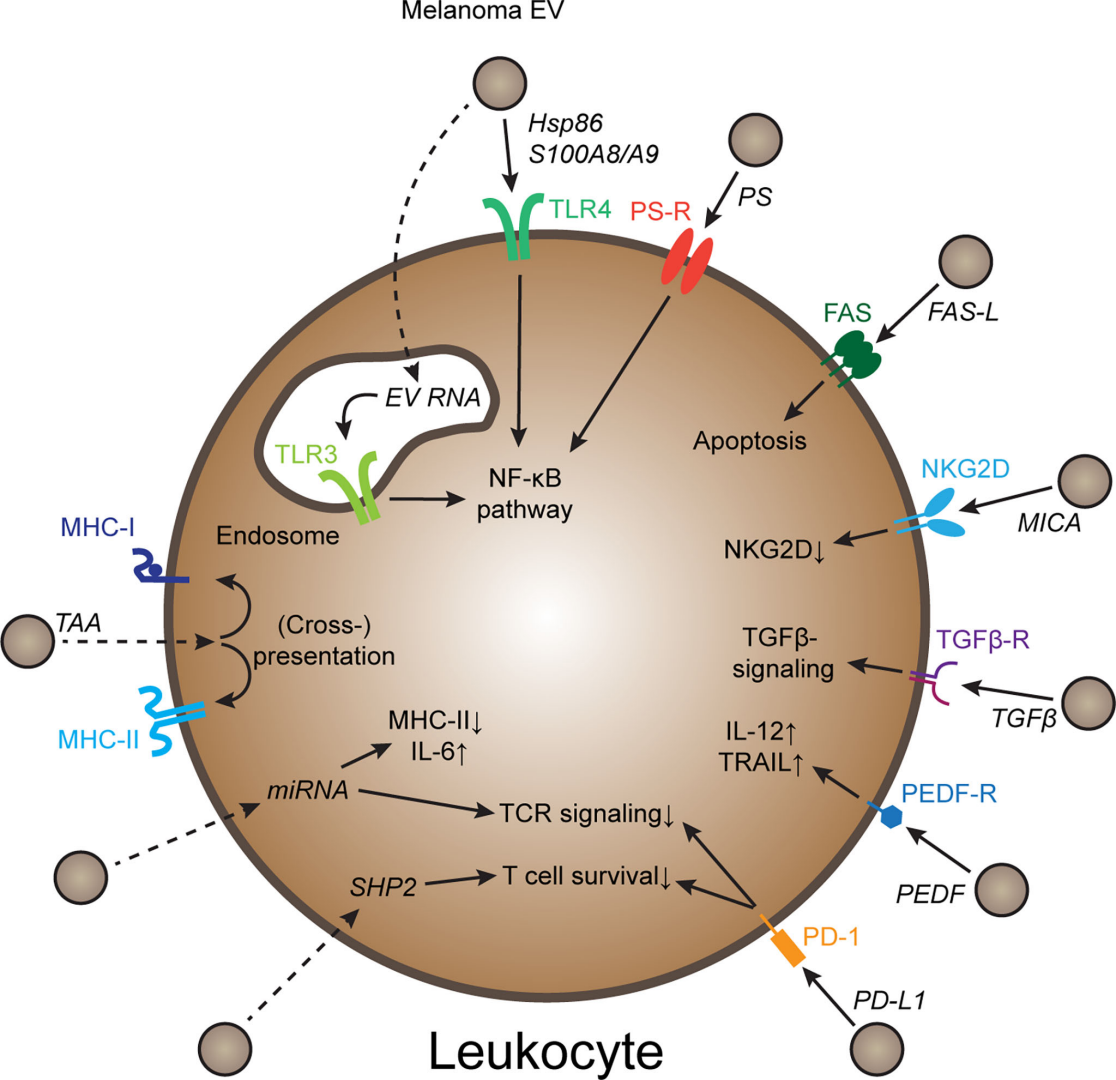
Mechanisms of melanoma EV-mediated immune cell regulation Schematic representation of several molecular mechanisms how melanoma-derived EVs can regulate immune cell behavior. Further explanations in the text. Dotted arrows indicate pathways that require EV uptake and release of EV cargo. PS, Phosphatidyl-serine; PS-R, PS-receptor; TAA, tumor-associated antigen; PEDF, pigment epithelium derived factor.

Melanoma is an aggressive cancer type prone to invade and metastasize, and melanoma-derived EVs have been implicated in progression (4). EV plasma levels in melanoma patients are increased compared to healthy individuals (5), and plasma EV signatures correlate with immunotherapy outcome (6–8), suggesting that melanoma-derived EVs might affect tumor immunity. Congruently, inhibition and activation of various immune cell types by melanoma EVs have been described (9). In this review, I focus specifically on mechanisms of EV-mediated immune regulation, such as innate immune-regulatory pathways, immune checkpoints, as well as transfer of tumor antigens and other immune-regulatory cargoes (**Figure 1**). Furthermore, current challenges in the field, including variability in experimental procedures, *in vitro* experiments failing to reflect natural EV biodistribution, and a lack of mechanistic *in vivo* studies are discussed.

## EV-mediated effects on the nuclear factor κ B (NF-κB) pathway

The NF-κB pathway is a highly conserved signal transduction pathway triggered by danger signals and inflammatory cytokines and regulating inflammatory responses in virtually every cell type. In melanoma, NF-κB activation has been linked to tumor initiation, progression and inflammation affecting tumor immunity and cancer stemness (10–12). Interestingly, several studies suggested that melanoma-derived EVs can de-regulate NF-κB signaling in immune cells. For example, EVs derived from the mouse melanoma cell lines B16F1 and B16F10 could activate NF-κB in RAW264.7 and primary macrophages, altering the release of inflammatory cytokines and chemokines (13, 14). More recently, melanoma-derived EVs isolated from plasma of melanoma patients using CSPG4-binding antibodies were shown to activate NF-κB in autologous, peripheral CD8+ T cells, while pharmacological NF-κB inhibition could reverse EV-mediated suppression of T cell proliferation (15). In contrast, Luong et al. found that melanoma-derived EVs induce SOCS3, a negative regulator of the NF-κB pathway, in bone marrow-derived monocytes (16). However, this effect was observed after prolonged stimulation (4 days) of monocytes. Thus, SOCS3 upregulation might have been due to a negative feedback response towards increased NF-κB activation.

Exactly how melanoma EVs trigger NF-κB activation in immune cells is not completely understood, but several pathways have been implied. For instance, EVs derived from human melanoma cell lines have been found to inhibit dendritic cell (DC) maturation *via* S100A8 and S100A9 proteins, which signal *via* TLR4, among others (17). TLR4 also mediated melanoma EV-induced upregulation of PD-L1 in immature myeloid cells *via* EV surface-associated Hsp86 (18). Furthermore, mouse melanoma cell-derived EVs could trigger TLR3 in bone marrow-derived DCs (19). Another potential mechanism how melanoma-derived EVs could affect NF-κB is *via* phosphatidyl-serine (PS). PS is usually excluded from the outer face of the plasma membrane but can be exposed on the surface of apoptotic cells and EVs. Importantly, PS has broad immune-inhibitory and tolerogenic functions, in part *via* regulation of NF-κB (20). Blockade of EV PS ameliorated TGF-β1 induction by B16F10-derived EVs in peritoneal macrophages (21) as well as the inhibition of primary human T cells by EVs derived from a melanoma xenograft (22). In contrast, EV PS has been reported to bind CD300 in bone-marrow derived DCs, preventing their activation of TLR3 (19).

## Melanoma EVs trigger apoptotic pathways

Melanoma-derived EVs might also impair tumor immunity by inducing apoptosis in immune effector cells. EVs isolated from human melanoma cell lines and from the serum of melanoma patients were shown to contain FasL and to induce apoptosis of patient-derived, melanoma-specific CD8+ T cells (23). In a follow-up study, melanoma EVs isolated from plasma of melanoma patients again consistently contained FasL as well as TRAIL, and FasL-blocking antibodies partially reduced EV-induced T cell apoptosis *in vitro* (15).

## NK-activating receptors: NKG2D and NKp30

NKG2D is an activating receptor expressed by NK cells and subsets of CD8+ T cells recognizing stress signals such as MICA and MICB on the surface of target cells. Secreted MICA on the other hand impairs NK and CD8+ T cell responses, and a human melanoma cell line expressing the MICA allele *008 has been shown to release MICA in association with EVs (24). MICA (but not MICB) was also present in melanoma EVs isolated from plasma of melanoma patients and these EVs downregulated NKG2D in primary human NK cells, suggesting functional impairment (15).

Activation of the innate pattern recognition receptor RIG-1 in human melanoma cell lines induced the release of EVs enriched for BAG6, a ligand for NKp30, another NK-activating receptor. In this case however, RIG-1-induced EVs activated the cytotoxicity of human NK cells *in vitro* and impaired primary tumor growth in a mouse model of melanoma (HCMel12) (25). This effect was not mediated by NKp30 though, since this receptor is not functional in mice (26). Instead, BAG6 promotes generation and cargo-loading of immune-stimulatory EVs released by stressed melanoma cells (27), thus explaining their immune-stimulatory function in mice lacking functional NKp30.

## EV-associated cytokines

Transforming Growth Factor β (TGF-β) is a pleiotropic cytokine that can inhibit tumor immunity in melanoma (28). Whether or not TGF-β acts at least partially *via* EVs is controversial. On the one hand, TGF-β was associated with EVs derived from the human melanoma cell line A375, and melanoma EV-induced inhibition of DC maturation could be reversed using TGF-β-blocking antibodies (29). On the other hand, melanoma EVs isolated from patient plasma were not enriched for TGF-β compared to non-melanoma EVs derived from the same patients, while EV-mediated suppression of T cell proliferation *in vitro* could still be blocked by anti-TGF-β (15). Thus, further studies are necessary to establish whether (respectively how) TGF-β is associated with melanoma EV surfaces, or whether it is induced *de novo* in recipient cells of melanoma EVs and thereby inhibits immune responses (21).

Interestingly, EVs derived from non-metastatic melanoma cell lines and plasma of patients with non-metastatic melanoma have been found to activate patrolling monocytes, macrophages, and NK cells and thereby to suppress melanoma lung metastasis *in vivo*. This effect was mediated by pigment epithelium-derived factor (PEDF), a secreted cytokine that was found to be associated with melanoma EV surfaces (30).

## Immune checkpoints: Do melanoma EVs inhibit T cells *via* PD-L1?

Immune checkpoints such as the CD80/86-CTLA and the PD-L1-PD-1 axes have recently gained much attention, since their therapeutic targeting elicits immune responses, particularly in melanoma that is characterized by a high mutation rate and occurrence of tumor (neo-) antigens. In melanoma, PD-L1 is expressed by immune, stromal, and tumor cells, enabling them to evade attacks by tumor antigen-specific CD8+ T cells in the tumor microenvironment. Importantly, several studies have shown that melanoma cells also release functional PD-L1 *via* EVs, potentially inhibiting T cells beyond the local microenvironment. Wei Guo’s team found surface PD-L1 on EVs isolated from several human and mouse melanoma cell lines, with higher levels in metastatic cell lines compared to non-metastatic ones (31). Furthermore, increased levels of PD-L1+ EVs could be detected in the plasma of patients with metastatic melanoma compared to healthy donors (31, 32). Functionally, EVs from melanoma cell lines could inhibit proliferation and cytotoxicity of CD8+ T cells *in vitro* in a PD-L1- (or PD-1-) dependent manner (15, 31, 32). Subsequently, this mechanism of EV-mediated T cell suppression was shown to depend on ICAM-1 on the EV surface (33). In the B16F10 model, melanoma EVs furthermore promoted primary tumor growth and systemically inhibited CD8+ T cell proliferation *in vivo* (31). Similar observations have been made using the B16F10 tail vein model of lung metastasis (32).

Somewhat contradictory to the above-mentioned studies, other groups failed to detect significant PD-L1 levels on melanoma-derived EVs. For instance, PD-L1 was neither detectable in melanoma cell line- and melanoma patient-derived EVs using mass spectrometry (34–36) nor was it enriched in melanoma-derived EVs compared to non-melanoma EVs isolated from the plasma of melanoma patients using a flow cytometry-based approach (15). Thus, the amount of PD-L1 in melanoma EVs might be low. Furthermore, PD-L1 blockade could not revert melanoma EV-mediated inhibition of cytokine production in patient-derived, NY-ESO-1-specific T cells unless it was combined with IL-10 blockade (37). Thus, the relevance of EV-associated PD-L1 compared to cellular PD-L1 for melanoma immunity and immunotherapy responsiveness is not entirely clear yet.

## Horizontal transfer of immune-modulatory cargo

Apart from ligands on the EV surface that trigger immune-regulatory receptors upon contact with or uptake by their recipient cells, EVs carry complex payloads including cytosolic proteins and nucleic acids such as miRNA, which they can transfer from one cell to another. Especially EV-mediated transfer of miRNAs has been studied to considerable extent, and there are reports that in the case of melanoma, this phenomenon can contribute to tumor-associated immune inhibition. Huber et al. identified several miRNAs (miR100, miR125b, miR146a and miR155), that, when transferred by melanoma EVs to monocytes, induced the conversion of monocytes into myeloid-derived suppressor cells (MDSCs), resulting in the downregulation of MHC-II and concomitant upregulation of IL-6 and CCL2 (38). More recently, Vignard and colleagues identified another set of miRNAs in human melanoma cell line-derived EVs that could downregulate TCR responses and effector functions in CD8+ T cells *in vitro* (39).

Another potential immune-regulatory cargo of melanoma EVs is SHP2. Wu et al. reported that EVs derived from mouse B16F0 melanoma cells contained both SHP2 protein and mRNA, and could transfer it to primary CD8+ T cells resulting in reduced T cell viability upon stimulation. However, it remained unclear whether this effect was due to mRNA or protein transfer, and significant SHP2 induction required rather high concentrations of EVs *in vitro* (40).

## EVs and tumor antigens

EVs contain a large variety of proteins derived from their donor cells, including potential tumor-associated antigens, which, if transferred to appropriate recipient cells, can be presented to the immune system. Indeed, more than two decades ago, it was shown that EVs derived from melanoma cell lines as well as malignant effusions of patients with metastatic melanoma contain classic melanoma antigens such as gp100 and Tyrp2 (41, 42). Subsequently, other studies confirmed these findings in human and mouse melanoma cell lines (23, 43). In addition, peptides derived from melanoma antigens might be transferred directly by EVs in association with MHC-I molecules (29, 44).

When transferred to immune-stimulatory antigen-presenting cells (APCs) such as mature DCs, melanoma EVs can induce tumor antigen presentation and T cell activation (41). However, if melanoma EVs deliver their cargo to tolerogenic APCs such as immature DCs, a state of “tumor tolerance” might ensue (45). Notably, melanoma EVs have been shown to inhibit DC maturation as discussed above (17, 29). Another tolergenic type of APC are lymphatic endothelial cells (LECs), particularly those lining lymph node (LN) sinuses. Previously, LN LECs have been shown to present self-antigens such as tyrosinase (which is also highly expressed in many melanomas) and inhibit T cells specific for it, possibly *via* PD-L1 which is constitutively expressed by LN LECs (46). We recently found that lymphatic PD-L1 can also impair tumor-specific T cell responses in melanoma-draining LNs (47), and that in the B16F10 melanoma model, EVs can transfer a model antigen from primary tumor cells to draining LN LECs, resulting in impaired CD8+ T cell responses (36). Thus, depending on the recipient cell type and state, melanoma EV-mediated transfer of tumor antigens can have either immune-stimulatory or tolerogenic effects.

## Location, location, location…

The majority of the studies discussed above investigated mechanisms of EV-mediated immune regulation using highly reductionistic *in vitro* systems, typically co-culturing immune cells directly with EVs isolated from cell culture or plasma samples. Although these approaches are useful to explore the overall immune-regulatory capacity of EVs, they do not reflect the aspect of EV biodistribution *in vivo*, which is very important regarding the question which immune cell types (and in which functional state) are most likely affected by melanoma EVs *in vivo*.

Within a growing melanoma, malignant cells release EVs into the surrounding interstitial space, enabling them to interact with tumor-infiltrating leukocytes in a paracrine manner. Melanoma EVs are also present in the blood, implying that they may affect immune responses systemically. Additionally, there is considerable evidence that melanoma EVs can be taken up by tumor-associated lymphatic vessels and are transported to draining LNs, important sites not only for early melanoma metastasis but also for the initiation (and inhibition) of tumor-specific immune responses (48, 49). In fact, initial lymphatic vessels, in contrast to blood vessels, are very permeable for particles up to 100-200 nm in diameter due to a specific junctional organization (“Button-like junctions”) between adjacent LECs. Furthermore, interstitial fluid dynamics facilitate EV transport towards lymphatic vessels within or around the tumor site (49). Indeed, in a seminal study in 2011, Joshua Hood and colleagues found that after interstitial injection, fluorescently labeled B16F10-derived EVs specifically and efficiently homed to draining LNs where they accumulated (50). Later on, several other groups confirmed efficient transport of both injected EVs as well as EVs released endogenously from primary B16 melanomas, to draining LNs in mice (34, 36, 51, 52). Using EV-associated luciferase activity for quantification, one of these studies furthermore found that the relative accumulation of melanoma-derived EVs was much higher in draining LNs compared to any other tissue in the body, including the blood (51). Similarly, melanoma-derived EVs were enriched in lymph-rich wound exudate collected after lymphadenectomy in melanoma patients in comparison to plasma, and lymph-borne EVs were larger than those from plasma, suggesting qualitative (and potentially, functional) differences between them (34, 35, 53).

Prominent uptake of melanoma EVs *in vivo* was noted especially in macrophages in the tumor microenvironment and in tumor-draining LNs (36, 51, 52), and this uptake has been suggested to prevent tumor growth-promoting B cell responses (51). While none of these studies found a significant uptake of melanoma EVs by other immune cell types, including DCs, NK cells, and T cells, melanoma EVs might still interact with them and affect their phenotype or function *via* cellular receptors as discussed above. Another cell type with a strong capacity to take up EVs are LN-resident LECs, and this uptake appears to depend on EV-associated integrins (36, 52). Recent advances in single-cell RNA sequencing allowed the identification of LN LEC subsets in human and mice (54, 55), and it were particularly LECs lining the floor of the subcapsular sinus that took up melanoma EVs (36), consistent with EV transport to LNs *via* afferent lymphatic vessels. In conclusion, melanoma derived EVs appear to have profound effects on tumor-draining LNs, primarily interacting with macrophages and LECs and thereby regulating adaptive T- and B-cell responses indirectly.

## Discussion

Clearly, melanoma-derived EVs have broad immune-regulatory capacities, triggering innate and checkpoint receptors in immune cells, transferring immune-modulatory cargoes including miRNAs, and delivering melanoma antigens to APCs, all of which may ultimately affect adaptive, tumor-specific immunity and immunotherapy responsiveness (**Figure 1**). However, there are considerable discrepancies between individual studies, both regarding the reported mechanisms as well as the direction of EV-mediated immune regulation. These divergent results are at least in part due to differences in the starting material (cell lines, individual patient samples) and experimental approaches such as EV isolation methods (resulting in varying EV subtypes and co-contaminants), EV dosing and choice of controls (e.g. empty liposomes or normal melanocyte-derived EVs). While there are efforts in the fields to standardize methods and quality controls (3), it will be an important challenge for future EV functional studies to identify experimental set-ups and conditions that yield the most clinically relevant results.

One important although experimentally challenging aspect in this regard is to validate *in vitro* observations of EV-mediated immune cell regulation using appropriate *in vivo* systems, particularly those reflecting endogenous EV release from a growing tumor. Those systems most faithfully represent “natural” EV doses, biodistribution, access to different tissues and immune cells within, and interplay between varying immune and stromal cells in response to melanoma-derived EVs. Also, patterns of cellular uptake of EVs are strikingly different *in vitro* and *in vivo*. However, such systems require elaborate approaches to allow tracking and modulation of endogenously secreted EVs, for instance using genetic EV tags and methods to modify EV (subset) release and/or composition *in vivo*. In the long run, such functional studies will be key to develop novel therapeutic approaches, targeting the release, uptake or effector functions of melanoma EVs, which could ultimately enhance endogenous as well as immunotherapy-induced immune responses in melanoma patients, for instance in unresectable disease or in a neo-adjuvant setting.

## Author contributions

LD wrote the manuscript and prepared the artwork. The author confirms being the sole contributor of this work and has approved it for publication.
