# Erratum: Mechanisms of extracellular vesicle-mediated immune evasion in melanoma

**DOI:** 10.3389/fimmu.2023.1178158

**Published:** 2023-03-13

**Authors:** Frontiers Production Office

**Affiliations:** Frontiers Media SA, Lausanne, Switzerland

**Keywords:** exosome, immune checkpoint, immunotherapy, melanoma, lymph node, metastasis, tumor immunity, extracellular vesicle (EV)

An omission to the funding section of the original article was made in error. The following sentence has been added: “Open access funding was provided by ETH Zurich”.

The original version of this article has been updated.

